# SPARQL Assist language-neutral query composer

**DOI:** 10.1186/1471-2105-13-S1-S2

**Published:** 2012-01-25

**Authors:** Luke McCarthy, Ben Vandervalk, Mark Wilkinson

**Affiliations:** 1 Providence Heart + Lung Institute at St. Paul's Hospital, University of British Columbia, Room 166 - 1081 Burrard St., Vancouver, BC, Canada V6Z 1Y6

## Abstract

**Background:**

SPARQL query composition is difficult for the lay-person, and even the experienced bioinformatician in cases where the data model is unfamiliar. Moreover, established best-practices and internationalization concerns dictate that the identifiers for ontological terms should be opaque rather than human-readable, which further complicates the task of synthesizing queries manually.

**Results:**

We present SPARQL Assist: a Web application that addresses these issues by providing context-sensitive type-ahead completion during SPARQL query construction. Ontological terms are suggested using their multi-lingual labels and descriptions, leveraging existing support for internationalization and language-neutrality. Moreover, the system utilizes the semantics embedded in ontologies, and within the query itself, to help prioritize the most likely suggestions.

**Conclusions:**

To ensure success, the Semantic Web must be easily available to all users, regardless of locale, training, or preferred language. By enhancing support for internationalization, and moreover by simplifying the manual construction of SPARQL queries through the use of controlled-natural-language interfaces, we believe we have made some early steps towards simplifying access to Semantic Web resources.

## Background

The health care and life science sectors have been some of the most enthusiastic adopters of Semantic Web technologies. The benefits of the RDF/OWL data model are well-understood by bioinformaticians who have too long had to deal with the problem of integrating data from multiple sources with wildly different underlying schema. These benefits are less obvious, however, to clinicians and researchers who merely see one mysterious query language (SQL) exchanged for another (SPARQL). Even a Semantic Web-savvy informatician can be daunted when faced with the challenge of querying an unfamiliar data source whose particular RDF vocabulary is initially unknown.

The issue is compounded by the growing use of opaque, semantic-free URIs for ontological classes and properties (OBO [1], SIO [2], CWA [3]). While the meaning of *rdf:type *or *dc:title *is relatively clear to the human reader, the meaning of, for example, *sio:SIO_010302 *is considerably harder to glean without looking up its ontological definition. This becomes particularly acute in the context of SPARQL queries. While often the subject and object portions of a SPARQL WHERE clause contain variables, and therefore are inherently human-readable, the predicates are usually explicitly specified in the query. Thus while:

?gene SIO:is_homologous_to ?gene

is quite clear to both the query composer and the human reader, the opaque equivalent

?gene SIO:010302 ?gene

is effectively meaningless to the reader, and absurdly difficult for the query composer to remember. Nevertheless, there are many valid reasons for designing ontologies using opaque identifiers, not the least of which is language neutrality.

RDF/XML provides built-in language neutrality by way of the *xml:lang *attribute; an ontology can therefore easily be internationalized by providing multiple *rdfs:label *or *rdfs:comment *properties with appropriate *xml:lang *attributes. However, even those projects who have, in principle, adopted language neutrality for their classes (e.g. OBO), most have not done so for their properties (OBO Relationship Ontology [4]). This is no-doubt due, at least in part, to the already discussed difficulty of composing SPARQL queries in which predicates have opaque identifiers. Nevertheless, it is crucial that we do not allow convenience to direct the development of a core global resource (the Semantic Web) and thus the problem should be solved at the level of the tools provided, rather than the resources themselves.

SPARQL Assist is a web application that facilitates the construction of SPARQL queries by providing context-sensitive type-ahead completion. In addition to assistance with basic syntax, ontological terms are indexed by their labels, using the *xml:lang *attribute to record the language of each label for each term. Ontological terms and their labels, are read on-the-fly from any ontology specified in a SPARQL FROM clause, but SPARQL Assist can also be configured to pre-load terms from particular ontologies or SPARQL endpoints, reducing the burden on the user to know the existence and location of all relevant vocabularies.

The entire query, as it is being constructed, is used to provide context for the type-ahead suggestions. Previously declared variables or known individuals (i.e. values) are suggested during type-ahead for the subject or object position of the WHERE clause. In the predicate position, indexed ontological predicates are suggested, with preference given to properties that an individual is known to have if this information is available from ontological indexing and/or other query clauses. Similarly, if a clause contains a variable that can ultimately be connected to a known individual in another part of the query, that connection is used to find the most likely properties in the current clause.

Together, these functionalities should simplify the manual construction of SPARQL queries by (a) making the query more similar to natural language, and (b) supporting any language in which the required terms have been labeled. Moreover, in so doing, SPARQL Assist also provides ontology designers more freedom to follow best-practices in ontology design and internationalization by reducing the barrier to query construction using opaque terms. SPARQL Assist thus represents one alternative, alongside visual query builders and faceted browsers, for helping unfamiliar users explore semantic data.

## Implementation

SPARQL Assist is implemented in JavaScript (jQuery) and is intended to be accessed through a browser. In its simplest configuration, SPARQL Assist preloads properties, individuals and namespaces that have been indexed in JSON format. It can also be deployed with a server-side Java component that will index OWL ontologies directly. These ontologies may be parsed a priori as part of the SPARQL Assist installation/configuration, or may be referred to in the FROM clause of a SPARQL query, in which case they are parsed dynamically at query time. In the future, as much computation as possible will be transferred to the client side to improve both performance and flexibility of deployment, but at present the limited support for OWL in JavaScript requires a server-side component for ontology parsing.

Our initial implementation of SPARQL Assist was undertaken in the context of creating queries that will be resolved by the Semantic Health and Research Environment (SHARE [5]), and we present here both the core SPARQL Assist software, as well as the extension specific to SHARE. SHARE is an advanced SPARQL query client built on top of the SADI Framework [6] for Semantic Web Services. SADI services attach properties to input OWL instances and these services are indexed in a central registry based on the properties they attach. SHARE maps the triple patterns presented in the WHERE clauses of a SPARQL query onto these indexed properties, in order to discover SADI Web Services capable of generating the required triples. The RDF data required to answer a given query is thus dynamically generated through the invocation of SADI services in response to the query being posed.

In the context of SPARQL Assist, the fact that properties do not exist at query composition time might be considered a barrier, since there is no pre-existing RDF Store to inspect for candidate properties and individuals. To compensate for this, the SADI extension to SPARQL Assist uses the SADI registry, in addition to any loaded ontologies, to suggest properties to be used in a query. As in the generic case, if a clause contains a named individual or a variable previously connected to an individual, this information is used to further refine the suggestions; in this case by filtering out properties generated by services that cannot accept a particular individual as input and highlighting properties generated by services that can. Thus, not only is the lack of a static triple store not a barrier to query composition, but the ability to construct a likely-successful query is, in fact, enhanced by utilization of the underlying SADI infrastructure.

Although it was designed with SHARE in mind, SPARQL Assist can be used with any SPARQL endpoint that can be queried from client-side JavaScript. Due to the limitations of cross-domain JavaScript requests, the endpoint must reside at the same domain name as the SPARQL Assist query form, or the endpoint must be able to deliver query results in JSONP format, or a suitable proxy must be configured. Sample configurations for common situations are available on the web [7].

## Results

The results of this software engineering project are best described by a demonstrative walk-through using our public SPARQL Assist interface available at [8]. In this demonstration we will be answering the question:

select the genes that participate in the human caffeine metabolism pathway and the proteins that they encode.

**Figure 1:**
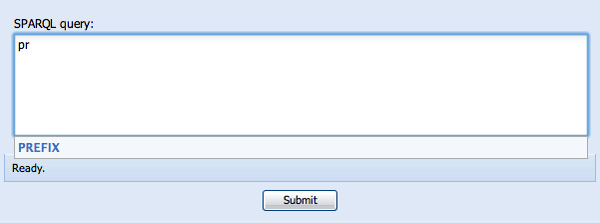
**Type-ahead suggestion for structural components of a SPARQL query**. The user has typed "pr..." and is being prompted that the appropriate syntactic element for this position in a SPARQL query is "PREFIX".

**Figure 2:**
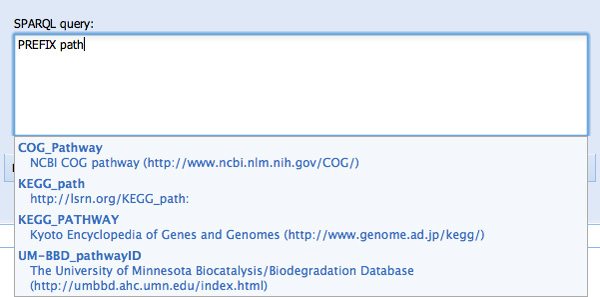
**Type-ahead suggestion for the URI of a namespace PREFIX**. The user has typed "path..." with the intention of finding the KEGG Pathway URI prefix. This is suggested for them as the third suggestion in the list. This relieves the user of the burden both recalling these URIs, and of correctly typing them.

SPARQL Assist helps query construction at all points, both with respect to correct SPARQL syntax, as well as the content of the query itself. This includes the specification of prefixes. Begin typing "PREFIX" and a type-ahead prompt appears indicating that PREFIX is the only valid option at this point (see Figure 1). Knowing that we are going to be using KEGG pathways, but not knowing what the correct URI prefix is, we then begin typing "path..." and are prompted with several options, one of which is the URI prefix for identifiers from the Kyoto Encyclopedia of Genes and Genomes (see Figure 2).

**Figure 3:**
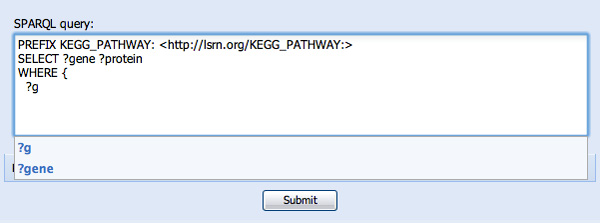
**Type-ahead selection of known query variables**. The user has already created two variables, "gene" and "protein" in their SELECT clause, and now is being prompted to reuse the "gene" variable in the WHERE clause under construction.

**Figure 4:**
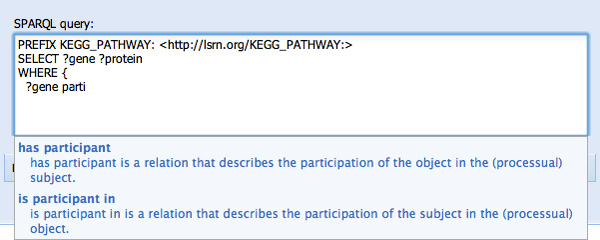
**Type-ahead selection of ontological predicates based on their human-readable label**. The user has started typing "parti...", with the intention of finding some predicate relating to "participation". Two possible predicates, "has participant" and "is participant in", are suggested, along with their human-readable descriptions.

**Figure 5:**
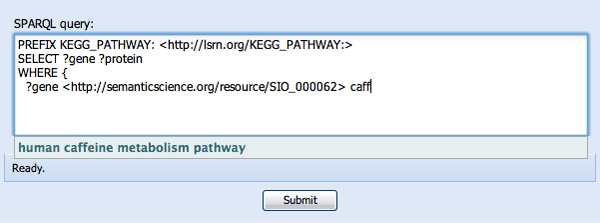
**Type-ahead selection of individuals by their human-readable name**. The user begins typing the word "caffeine" and is prompted by "human caffeine metabolism pathway", a specific pathway in the KEGG database. Selection of this pathway results in the insertion of the corresponding URI into the SPARQL query.

Several steps later, after specification of the SELECT variables (not shown), SPARQL Assist has a number of features aimed at simplifying the construction of the WHERE clause. First, previously-typed variables are prompted. For example, typing "?g" prompts the user with their previously entered "?gene" variable (see Figure 3). Knowing (*a priori*) the vernacular that genes "participate in" metabolic pathways, typing "parti..." results in two choices of predicate "has participant", and "is participant in", along with their human-readable descriptions to help determine which predicate would be the correct one (see Figure 4). In this case, "is participant in" is selected, since genes are participants in pathways. The opaque URI of the corresponding predicate (*SIO:SIO_000062 *from the Semantic Science Integrated Ontology) is added to the SPARQL query as a result of this selection (see Figure 5).

**Figure 6:**
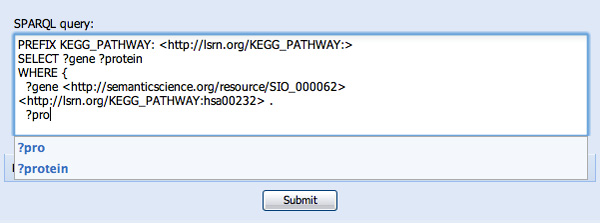
**Replacement of named individuals by their URI**. In this figure, "human caffeine metabolism pathway" has been replaced by its URI http://lsrn.org/KEGG_PATHWAY:hsa00232. The user is now being prompted to use the previously-defined "protein" variable for their next clause.

With an interest in genes involved in caffeine metabolism, and not knowing which KEGG pathway this corresponds to, we then begin typing "caff..." in the object-position of the WHERE clause. SPARQL assist has pre-indexed the names and definitions of KEGG pathway URIs, and thus prompts us with "human caffeine metabolism pathway" as a type-ahead option (see Figure 5). Selecting this option inserts the URI *KEGG:hsa00232 *in the object position, corresponding to the identifier for this pathway, and the first WHERE clause is complete (see Figure 6).

**Figure 7:**
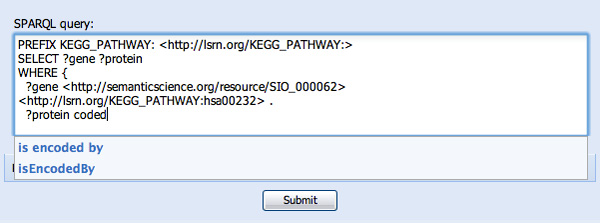
**Naming collisions are observed**. In this figure, the user is looking for a predicate related to gene coding. They are presented with two seemingly identical predicates, neither of which has a description. In cases where independent ontologies use the same term and do not define that term, it is not possible for the software to automatically infer equivalence. As such, both terms are presented to the user and they must make a choice based on minimal information.

**Figure 8:**
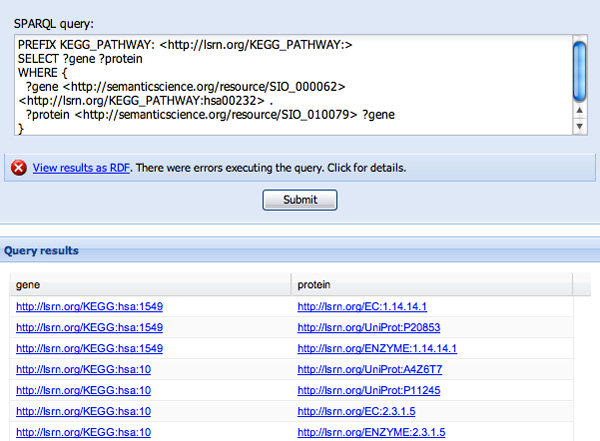
**Query completion and execution**. The predicate "is encoded by" has been replaced by its URI http://semanticscience.org/resource/SIO_010079 the final variable was added to the second clause, and the query was executed with the results shown.

In constructing the second WHERE clause, we will reveal some limitations of the approach. After being prompted for "?protein" in response to "?pro..." (see Figure 6) we then want to determine which genes code for these proteins. Typing "code" reveals two, seemingly identical, options, neither of which has a definition - "is encoded by" and "isEncodedBy" (see Figure 7). This is because these two predicates appeared in the ontologies pre-indexed by SPARQL Assist and had no further description. Since it is not possible to determine with any certainty if they are truly redundant, or which online resources will use which predicate, both are presented, and the user must make a choice. In Figure 8 we have selected "is encoded by", and the corresponding URI http://semanticscience.org/resource/SIO_010079 has been added to the query. We complete the query by adding the final ?gene variable, and the query results are shown in the same figure.

Three additional features of the SPARQL Assist software, and its SADI extension -- internationalization, dynamic ontology indexing, and dynamic predicate-validation -- will be demonstrated by a second walk-through. In this example, we attempt to resolve the same query as above, but will construct the query clauses slightly differently to allow these additional features to be revealed (in SPARQL, equivalent queries can be constructed in a wide variety of ways, particularly when the desired predicates have an *owl:inverse *predicate).

**Figure 9:**
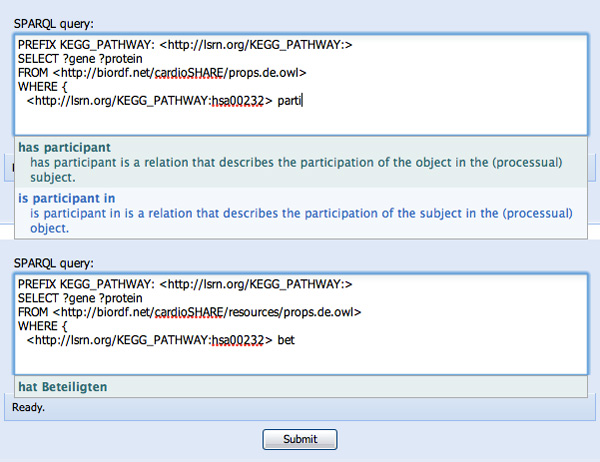
**SADI registry lookups and internationalization**. This figure shows support for internationalization as well as the additional functionality of SPARQL Assist provided by the SADI extension. When a named individual is present in a query, the SADI extension to SPARQL Assist utilizes the semantic type of this individual to initiate a search of the SADI registry for Services that could consume this individual as input. The predicates created by those services are highlighted in green in the type-ahead prompt, providing visual assurance that selection of those predicates is "semantically meaningful" and is likely to resolve successfully during query execution. In the top panel, the user has started typing (in English) "parti...", and the predicate "has participant" from the type-ahead choices is highlighted in green, indicating that a SADI Service is available that will generate that predicate based on that input individual. In the lower panel, at the same point in the query, the user has started typing (in German) "Bet...", and is provided with the predicate "hat Beteiligten". Since "hat Beteiligten" and "has participant" are alternative labels for the same predicate URI (http://semanticscience.org/resource/SIO_000132), this predicate is also highlighted in green indicating that there is a SADI service capable of resolving it.

Figure 9 shows the initial phases of query construction already completed as above, this time with the addition of a FROM clause. When confronted with a FROM URI, SPARQL Assist will immediately retrieve and index the resulting ontology, extracting predicates and named individuals from that ontology. In this example, the *props.de.owl *ontology contains a variety of German-language extensions for predicates defined by the SIO, and individuals from KEGG. When constructing the first WHERE clause in this case, we have decided to begin with the KEGG caffeine metabolism pathway. This was selected exactly as in Figure 5 above, but using the German equivalent "Stoffwechselweg Koffein menschlichen" as the individual name (not shown). With an explicit individual as the starting-point, the SADI extension to SPARQL Assist is able to utilize the semantics of the SADI Web Services registry to enhance subsequent type-ahead suggestions. To find the genes that participate in this KEGG pathway, we begin typing "parti..." in the predicate position of this WHERE clause. As before, SPARQL Assist prompts us with a variety of suggestions, however one of these suggestions has been highlighted in green (see Figure 9 upper panel). This highlighting indicates that SPARQL Assist has discovered SADI Web Services capable of fulfilling that clause of the query, given that individual's semantic type, thus giving the user more security that their query will be answerable. Because the German-language ontology has been loaded, we could equally have begun typing "Bet...", and been prompted by the German equivalent predicate "hat Beteiligten", also highlighted in green (see Figure 9 lower panel).

**Figure 10:**
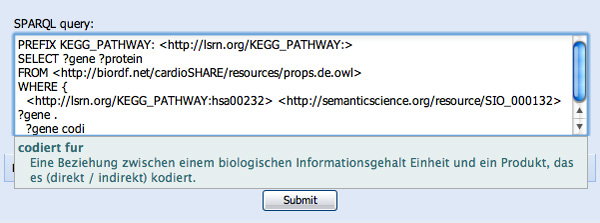
**Additional utilization of SADI registry semantics**. The German-language "codiert für" (English "codes for") is being suggested, and is highlighted in green. The green highlighting indicates that the SPARQL Assist software, after the previous SADI Registry lookup (see Figure 9) knows what semantic datatype will be contained in the variable ?gene after invocation of that SADI service, and is therefore able to determine that another SADI service is available that will be able to consume individuals from ?gene and attach the "codiert für" predicate to them.

**Figure 11:**
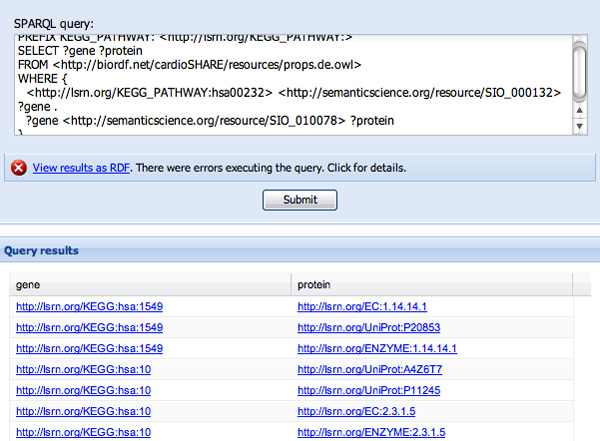
**Query completion and solution**. In this figure, "codiert fur" has been properly converted to its SIO URI. Note also that the query results are identical to those in Figure 8, demonstrating that these queries, regardless of language chosen, are equivalent.

We complete the first clause and continue to the next, where we wish to find the proteins that are encoded by a given gene. We begin the second WHERE clause with "?gene", and then (in the German language) attempt to find the proteins that the genes "codiert für" (see Figure 10). Importantly, SPARQL Assist is able to suggest predicates based on the semantic type of the data that will fill the ?gene variable. It is able to do this because, during the previous SADI registry query for the "has participant" predicate, it determined the output data-type of that SADI service and assigned that data-type to ?gene. As such, SPARQL Assist is now able to indicate (through green highlighting) that there is a SADI service that attaches the "codiert für" predicate to the individuals that will, at execution-time, fill the ?gene variable. Finally, the "codiert für" predicate is selected and replaced by the appropriate URI, and query construction continues through to resolution (see Figure 11).

## Discussion

The two walk-through scenarios above reveal the behaviours of the SPARQL Assist software and demonstrate what we believe is an environment that assists query construction not only by "naïve" end-users, but also by experienced informaticians already comfortable with the SPARQL language. While these behaviours (e.g. type-ahead) are not themselves novel, the utility comes in the way they have been applied, particularly with respect to dynamic indexing of ontological terms, predicates, and individuals, and explicit support for internationalization.

We are aware that it may seem unsustainable and/or excessive to index individuals, such that "real things" (molecules, structures, genes, etc) can be referred-to during construction of the query and resolved by SPARQL Assist to their native identifiers, but we don't think this is necessarily the case. First, we believe it is crucial to do so since (as with browser bookmarks for URLs) we cannot and should not expect users to know or remember the URI of their data-of-interest. Second, the amount of storage required to achieve this (a URI and it's various labels) is minimal and lookups over this data can be quite rapid. Third, we anticipate that SPARQL Assist will be deployed by specific communities or on specific portals, where the individuals of greatest interest to that community can be anticipated and selectively indexed to minimize lookup time and index size.

SPARQL Assist does not aim to be a query interface that understands natural language. It relies on ontology authors creating "obvious" labels for their predicates and, even in that case, a user will often need to try several "words" before discovering the phrase that the ontology author used. However, we believe that this is nevertheless better than the status quo and, moreover, makes it easier for ontology authors to justify following best-practices of ontology design and internationalization by reducing the resulting burden of complexity placed on their end-users.

There are some areas where the SPARQL Assist interface can be further improved. In particular, while the query construction process itself is guided by "natural language", the interface immediately converts this into SPARQL clauses with opaque identifiers; thus, the final query in SPARQL Assist is just as unreadable as any traditional SPARQL query. This implementation barrier results from limitations in the JavaScript language: the content of a Web text-box cannot be marked-up and thus SPARQL query clauses cannot be individually referenced and linked to an external, non-SPARQL representation. We would welcome participation of any community members who might have a solution to this problem!

## Conclusions

SPARQL Assist provides prototype solutions for two important problems. First, to hasten the uptake of Semantic Web technologies, it is important to improve access to, and usability of, Semantic Web resources for the lay-end-user while still maintaining best-practices in the way these resources are modeled. Opaque identifiers for both classes and properties are important, as they allow us to avoid "churn" as an ontology evolves over time. We must therefore support the end-user in constructing queries over resources formatted in this way. Second, the Semantic Web is intended to be a global resource, of use to all. As such, a respect for internationalization is also critical, even at these early stages in Semantic Web evolution. We believe that SPARQL Assist provides motivation to more widely adopt what are clearly best-practices in Semantic Web data provision.

## Availability and requirements

**Project name**: SPARQL Assist

**Project home page**: http://code.google.com/p/sadi/

**Operating system(s)**: Platform independent

**Programming language**: Java

**License**: New BSD

## List of abbreviations

CWA: Concept Web Alliance; JSON: JavaScript Object Notation; JSONP: JavaScript with Padding; KEGG: Kyoto Encyclopaedia of Genes and Genomes; OBO: Open Biomedical Ontologies; OWL: Web Ontology Language; RDF: Resource Description Framework; SADI: Semantic Automated Discovery and Integration; SHARE: Semantic Health And Research Environment; SIO: Semantic Science Integrated Ontology; SPARQL: SPARQL Protocol and RDF Query Language; SQL: Structured Query Language; URI: Uniform Resource Identifier; URL: Uniform Resource Locator; XML: Extensible Markup Language.

## Competing interests

The authors declare that they have no competing interests.

## Authors' contributions

LM wrote the SPARQL Assist software and wrote significant portions of this manuscript. BV invented and implemented the optimizer algorithm of SHARE allowing SPARQL queries to be efficiently converted to Web Service workflows for this software demonstration and assisted with portions of this manuscript. MW conceived of the SADI project, together with LM conceived of the SPARQL Assist concept, and wrote the majority of this manuscript.
